# Atrial High-Rate Event Incidence and Predictors in Patients With Permanent Pacemaker Implantation

**DOI:** 10.3389/fcvm.2021.728885

**Published:** 2021-10-11

**Authors:** Jian Hua Chen, Guo Yao Chen, Hong Zheng, Quan He Chen, Fa Yuan Fu, Fei Long Zhang, Liang Long Chen, Wei Wei Wang

**Affiliations:** ^1^Department of Cardiology, Fujian Provincial Institute of Coronary Disease, Fujian Heart Medical Center, Fujian Medical University Union Hospital, Fuzhou, China; ^2^Union Clinic Medical College, Fujian Medical University, Fuzhou, China

**Keywords:** atrial high-rate events, pacemaker, sick sinus syndrome, percentage of atrial pacing, cardiology

## Abstract

**Objective:** The present study aims to investigate the incidence and predictors of atrial high-rate events (AHREs) in patients with permanent pacemaker implants.

**Methods:** A total of 289 patients who were implanted with a dual-chamber pacemaker due to complete atrioventricular block or symptomatic sick sinus syndrome (SSS) and had no previous history of atrial fibrillation were included in the present study. AHREs are defined as events with an atrial frequency of ≥175 bpm and a duration of ≥5 min. The patients were divided into two groups according to whether or not AHREs were detected during the follow-up: group A (AHRE+, *n* = 91) and group N (AHRE–, *n* = 198).

**Results:** During the 12-month follow-up period, AHREs were detected in 91 patients (31.5%). The multivariate Cox regression analysis revealed that patient age [odds ratio [OR] = 1.041; 95% confidence interval [CI], 1.018–1.064; and *P* < 0.001], pacemaker implantation due to symptomatic SSS (OR = 2.225; 95% CI, 1.227–4.036; and *P* = 0.008), and the percentage of atrial pacing after pacemaker implantation (OR = 1.010; 95% CI, 1.002–1.017; and *P* = 0.016) were independent AHRE predictors.

**Conclusion:** The AHRE detection rate in patients with pacemaker implants was 31.5%. Patient age, pacemaker implantation due to symptomatic SSS, and the percentage of atrial pacing after pacemaker implantation were independent AHRE predictors.

## Introduction

Cardiac implantable electronic devices (CIEDs) with atrial leads can detect atrial arrhythmia events whether or not the patient has symptoms (1). The European Society of Cardiology 2020 Guidelines for the Diagnosis and Management of Atrial Fibrillation (2) defines atrial high-rate events (AHREs) as events with an atrial frequency of ≥175 bpm and a duration of ≥5 min. AHREs are associated with an increased risk of clinical atrial fibrillation (AF) (3), ischemic stroke or systemic embolism (4), and cardiovascular death (5). A relevant study revealed that patient age, previous AF history, white blood cell count, and an increased C-reactive protein level were associated with AHRE occurrence (6). CIEDs include the permanent pacemaker (PPM), implantable cardioverter-defibrillator (ICD), and cardiac resynchronization therapy (CRT) instruments. However, there is no definite conclusion regarding the factors related to AHREs after PPM implantation. The present study aims to investigate the incidence and predictive factors of AHREs after PPM implantation in order to provide a reliable basis for the clinical identification of patients with a high AHRE risk.

## Methods

Subjects: The present study enrolled a total of 377 patients with a complete atrioventricular block (AVB) and symptomatic sick sinus syndrome (SSS) who met the class I or class IIa pacemaker implantation indications in accordance with the guidelines for the treatment of pacemakers with abnormal cardiac rhythm (7) and underwent dual-chamber PPM implantation. Of these patients, 308 met the inclusion and exclusion criteria.

Inclusion criteria: (1) Patients aged ≥18 years; (2) patients with no clinical evidence of a past AF attack; (3) patients with a ventricular pacing lead implantation site in the right ventricular septal plane and atrial pacing lead implantation site in the right atrial appendage; and (4) patients in whom the implanted pacemaker could diagnose and record atrial arrhythmia.

Exclusion criteria: (1) Patients aged <18 years; (2) patients with clinical evidence of AF attacks before pacemaker implantation; (3) patients with a history of transient ischemic attack, stroke, and myocardial infarction; (4) patients with chronic rheumatic valvular heart disease, congenital heart disease, or hypertrophic cardiomyopathy; (5) patients with a ventricular pacing lead implantation site not in the right ventricular septal plane (e.g., the right ventricular apex, His bundle area, or left bundle branch area) or an atrial pacing lead implantation site not in the right atrial appendage (e.g., the right atrial septal plane); and (6) patients who had a VVI pacemaker implanted during the operation due to the atrial lead pacing parameters not being ideal.

Pacemaker lead implantation methods: The fixed pacing leads were implanted through the subclavian vein or axillary vein. The ventricular pacing lead implantation site was the right ventricular septal plane, and the atrial pacing lead implantation site was the right atrial appendage. After the operation, the pacemaker was set to the DDD (R) mode, and the pacemaker's detecting, recording, and atrial arrhythmia storing functions were started. Medtronic pacemakers have a default AHRE frequency of 175 bpm, and Abbott pacemakers have a default rate of 180 bpm. After implantation, the Abbott pacemaker AHRE frequency was programmed to 175 bpm; during the follow-up, the pacemaker could indicate AHRE presence as well as the length of each event. An AHRE lasting ≥5 min was considered AHRE+; otherwise, it the AHRE was considered AHRE–.

Drug treatment after PPM implantation: If necessary, clinicians could freely choose renin-angiotensin system inhibitors (RAS-I) (e.g., angiotensin-converting enzyme inhibitors or angiotensin receptor blockers, β receptor blockers, and statins) or antiarrhythmic drugs (e.g., propafenone, amiodarone, sotalol, and diltiazem) for treatment, depending on whether the patient had complications of hypertension, coronary heart disease, diabetes, hyperlipidemia, or atherosclerosis.

Follow-up: The patients were followed up with for 12 months at 1 week after the operation and every 1–3 months after that. The specific follow-up included: (1) AHRE occurrence and the time from PPM implantation to the first AHRE occurrence; (2) the atrial pacing percentage (AP%) and ventricular pacing percentage (VP%); (3) AHRE type clarification via a 12-lead synchronous recording electrocardiography or 12-lead 24-h dynamic electrocardiography examination in patients with positive signs.

Research grouping: The patients were divided into two groups according to whether or not AHREs were detected by pacemakers during the follow-up: group A (AHRE+, *n* = 91) and group N (AHRE–, *n* = 198).

Statistical methods: All data analyses were based on the intention-to-treat analysis principle. Count data were expressed in percentage (%) and compared between the two groups using Pearson's Chi-square test or Fisher's exact test. Measurement data were first evaluated using a non-parametric test (Kolmogorov–Smirnov test); data in normal distribution were expressed as mean ± standard deviation and compared between the groups using *t*-tests, while data not in normal distributions were expressed as the median [25% percentile, 75% percentile, M[Q25, Q75]] and compared between the groups using non-parametric tests. The multivariate analysis was performed using logistic regression models, and the independent predictors of AHREs and endpoint events were analyzed and screened using Cox's proportional hazards model. The receiver operator characteristic (ROC) curve was used to find the best independent predictor diagnostic cut-off value, and the differences in post-operative AF-free and endpoint event-free survival time were analyzed using the Kaplan–Meier survival analysis and evaluated using log-rank (Mantel–Cox) tests. All data analyses were conducted using the SPSS software 26.0. A (two-sided) *P*-value of < 0.05 was considered statistically significant.

## Results

A total of 308 patients who met the inclusion and exclusion criteria and completed the operation were included in the 12-month follow-up, which was completed by 289 patients; 19 patients [7 patients in group A and 12 patients in group N [*P* = 0.596]] either withdrew or were lost to the follow-up. Of these 289 patients, AHREs were detected in 91 [31.5%, 47 males [51.6%]], 76 of whom (83.5%) underwent 12-lead synchronous electrocardiography or 12-lead 24-h Holter after AHRE detection; of these, 71 (93.4%) were diagnosed with AF, atrial flutter (AFL), or both, and 3 (3.9%) were diagnosed with paroxysmal supraventricular tachycardia. An electrophysiological examination confirmed atrioventricular nodal re-entrant tachycardia, and a radiofrequency ablation was successfully carried out. A total of 2 patients (2.6%) had negative examination results.

Of the 289 patients who completed the follow-up, 91 were in group A and 198 were in group N. The patient age was significantly higher in group A (71.3 ± 9.5) than in group N (66.0 ± 11.7) (*P* < 0.001), and the symptomatic SSS diagnosis proportion was higher in group A (83.5%) than in group N (56.1%) (*P* < 0.001). There were no significant differences in other baseline data between the two groups (Table 1).

**Table 1:** Comparison of baseline data of two groups before PPM implantation.

**Groups**		**Group A**	**Group N**	***P***
		**(AHRE+, *n* = 91)**	**(AHRE–, *n* = 198)**	
Age (year)		71.9 ± 9.2	66.3 ± 11.7	0.000
Male (cases)		47 (51.6%)	103 (52.0%)	0.953
BMI (kg m^−2^)		23.3 (22.3–25.1)	23.1 (21.4–25.1)	0.302
Diagnosis	Atrioventricular block (cases)	15 (16.5%)	87 (43.9%)	0.000
	Sinus node dysfunction (cases)	76 (83.5%)	111 (56.1%)	
Hypertension (cases)		44 (48.4%)	91 (46.0%)	0.705
Diabetes (cases)		13 (14.3%)	30 (15.2%)	0.848
Coronary heart disease (cases)		8 (8.8%)	14 (7.1%)	0.608
Peripheral vascular diseases (cases)		3 (3.3%)	3 (1.5%)	0.324
History of TIA/stroke (cases)		4 (4.4%)	7 (3.5%)	0.723
CHA_2_DS_2_-VASC score	0	7 (7.7%)	28 (14.1%)	0.241
	1	22 (24.2%)	51 (25.8%)	
	>2	62 (68.1%)	119 (60.1%)	
NYHA cardiac function classification (cases)	I	56 (61.5%)	144 (72.7%)	0.234
	II	26 (28.5%)	43 (21.7%)	
	III	8 (8.8%)	9 (4.5%)	
	IV	1 (1.1%)	2 (1.0%)	
Pacemaker manufacturer	Abbott	47 (51.6%)	114 (57.6%)	0.346
	Medtronic	44 (48.4%)	84 (42.4%)	
Manufacturer of pacemaker checked by	LVED (mm)	49.2 ± 5.9	48.5 ± 4.6	0.277
transthoracic echocardiography	LVES (mm)	31.3 (28.0–34.0)	30.2 (27.8–32.8)	0.173
	LA (mm)	38.0 (33.8–41.9)	35.6 (32.8–38.9)	0.009
	LVPW (mm)	9.8 (9.2–11.2)	9.6 (8.9–10.4)	0.046
	IVS (mm)	10.4 (9.4–11.8)	10.1 (9.3–11.2)	0.293
	LVEF (%)	66.2 (62.6–70.0)	67.5 (62.2–71.2)	0.231
	LVWI (g m^−2^)	110.8 (96.0–137.6)	104.5 (95.0–119.7)	0.023
	E/E′	13.2 (10.8–19.0)	12.2 (10.0–15.6)	0.080
	E/A	1.0 (0.8–1.4)	0.9 (0.7–1.3)	0.301

*MI, body mass index; NYHA, New York Heart Association; LVED, left ventricular end-diastolic diameter; LVES, left ventricular end-systolic diameter; LA, left atrial diameter; LVPW, left ventricular posterior wall thickness; IVS, interventricular septal thickness; LVEF, left ventricular ejection fraction; LVWI, left ventricular mass index*.

The left atrium was larger in group A [38.0 mm [33.8–41.9 mm]] than in group N [35.6 mm [32.8–38.9 mm]] (*P* = 0.009), the left ventricular posterior wall was thicker in group A [9.8 mm [9.2–11.2 mm]] than in group N [9.6 mm [8.9–10.4 mm]] (*P* = 0.046), and the left ventricular mass index was higher in group A [110.8 g/m^2^ [96.0–137.6 g/m^2^]] than in group N [104.5 g/m^2^ [95.0–119.7 g/m^2^]] (*P* = 0.023). There were no significant differences in the other transthoracic echocardiography parameters between the two groups (Table 1).

The AP% was higher in group A [68.0% [42.0–89.0%]] than in group N [38.5% [13.9–70.0%]] (*P* < 0.001). There was no difference in VP% between the two groups [8.3% [1.1–71.0%] in group A and 19.5% [2.2–97.8%] in group N] (*P* = 0.060, Table 2).

**Table 2:** Comparison of post-operative data between two groups after PPM.

**Groups**	**Group A (AHRE+**,	**Group N (AHRE–**,	***P***
	***n* = 91)**	***n* = 198)**	
**Pacing rate after PPM**			
AP (%)	68.0 (42.0–89.0)	38.5 (13.9–70.0)	0.000
VP (%)	8.3 (1.1–71.0)	19.5 (2.2–97.8)	0.060
**Drug use after PPM**			
ACE-I/ARB (cases)	28 (30.8%)	48 (24.2%)	0.242
Post-operative use of β-receptor blockers (cases)	33 (36.3%)	28 (14.1%)	0.000
Diltiazem	4 (4.4%)	13 (6.6%)	0.466
CCB (cases)	19 (20.9%)	41 (20.7%)	0.973
Post-operative use of statins (cases)	43 (47.3%)	86 (43.4%)	0.544
Post-operative use of antiarrhythmic drugs (cases)	18 (19.8%)	12 (6.1%)	0.005
Amiodarone	10	7	
Propafenone	7	3	
Sotalol	1	2	

*AP, atrial pacing; VP, ventricular pacing; ACE-I, angiotensin converting enzyme inhibitor; ARB, angiotensin II receptor blocker; CCB, calcium channel blocker*.

The proportion of patients who were treated with β-receptor blockers was higher in group A [33/91 [36.3%]] than in group N [28/198 [14.1%]] (*P* < 0.001), and the proportion of patients who were treated with an oral administration of antiarrhythmic drugs was higher in group A [18/91 [19.8%]] than in group N [12/198 [6.1%]] (*P* = 0.001). There were no significant differences in the proportions of patients who were treated with oral administration of RAS-I, calcium channel blockers, or statins between the two groups (Table 2).

The univariate logistic regression analysis revealed that patient age, diagnosis, left atrium size, left ventricular mass index, AP%, VP%, oral administration of β-receptor blockers, or Antiarrhythmic drugs (AADs) treatment were AHRE risk factors (Table 3), and the multivariate logistic regression analysis revealed that patient age [odds ratio [OR] = 1.060; 95% confidence interval [CI], 1.027–1.093; and *P* < 0.001], symptomatic SSS diagnosis (OR = 4.550; 95% CI, 1.634–12.669; and *P* = 0.004), AP% (OR = 1.018; 95% CI, 1.007–1.029; and *P* = 0.001), and oral administration of β-receptor blockers (OR = 2.520; 95% CI, 1.275–4.981; and *P* = 0.008) were AHRE risk factors (Table 4). The ROC curve analysis revealed that the best age diagnostic cut-off value was 65 years [area under the curve [AUC] = 0.641; sensitivity = 74.7%; specificity = 47.5%; and *P* < 0.001] and the best AP% diagnostic cut-off value was 53.5% (AUC = 0.692; sensitivity = 69.2%; specificity = 63.1%; and *P* < 0.001) (Table 5).

**Table 3:** Binary univariate logistic regression analysis of AHREs.

	**B**	**Standard error**	**Wald**	**Significance**	**Exp (B)**	**95% CI**	
						**Lower limit**	**Upper limit**
Gender	0.015	0.253	0.003	0.953	0.985	0.600	1.619
Age	0.050	0.013	14.829	0.000	1.051	1.025	1.078
BMI	0.020	0.038	0.271	0.603	1.020	0.946	1.100
Diagnosis	1.379	0.317	18.956	0.000	3.971	2.135	7.388
Hypertension	0.096	0.254	0.143	0.705	1.101	0.670	1.810
Diabetes	−0.069	0.359	0.037	0.848	0.933	0.462	1.887
Coronary heart disease	0.236	0.463	0.261	0.609	1.267	0.512	3.136
History of TIA/stroke	0.227	0.640	0.126	0.227	0.640	0.126	4.398
Peripheral vascular diseases	0.796	0.827	0.927	0.336	2.216	0.439	11.197
CHA_2_DS_2_-VASc	−0.350	0.268	1.711	0.191	0.705	0.417	1.191
NYHA	0.356	0.189	3.556	0.059	1.428	0.986	2.069
LVED	0.027	0.025	1.182	0.277	1.028	0.978	1.080
LVES	0.047	0.026	3.377	0.066	1.048	0.997	1.103
LA	0.045	0.021	4.590	0.032	1.046	1.004	1.089
LVPW	0.111	0.083	1.798	0.180	1.118	0.950	1.315
IVS	0.052	0.060	0.737	0.391	1.053	0.936	1.186
LVEF	−0.020	0.017	1.358	0.244	0.980	0.947	1.014
LVWI	0.009	0.004	4.434	0.035	1.009	1.001	1.017
E/E′	0.028	0.020	1.917	0.166	1.028	0.988	1.070
E/A	0.133	0.276	0.234	0.629	1.142	0.666	1.960
Pacemaker manufacturer	0.239	0.254	0.886	0.346	1.271	0.772	2.092
AP	0.022	0.004	25.087	0.000	1.022	1.014	1.031
VP	−0.006	0.003	4.096	0.043	0.994	0.987	1.000
ACEI/ARB	0.322	0.281	1.309	0.253	1.380	0.795	2.394
β-receptor blockers	1.240	0.299	17.240	0.000	3.454	1.924	6.202
Statins	0.154	0.254	0.368	0.544	1.167	0.709	1.920
CCB	0.010	0.312	0.001	0.973	1.011	0.548	1.862
AAD	1.341	0.397	11.380	0.001	3.822	1.754	8.329

*BMI, body mass index; NYHA, New York Heart Association; LVED, left ventricular end-diastolic diameter; LVES, left ventricular end-systolic diameter; LA, left atrial diameter; LVPW, left ventricular posterior wall thickness; IVS, interventricular septal thickness; LVEF, left ventricular ejection fraction; LVWI, left ventricular mass index; AP, atrial pacing; VP, ventricular pacing; ACE-I, angiotensin converting enzyme inhibitor; ARB, angiotensin II receptor blocker; CCB, calcium channel blocker; AAD, antiarrhythmic drugs*.

**Table 4:** Binary multivariate logistic regression analysis of AHREs.

	**B**	**Standard error**	**Wald**	**Significance**	**Exp (B)**	**95% CI**	
						**Lower limit**	**Upper limit**
Age	0.058	0.016	13.456	0.000	1.060	1.027	1.093
Diagnosis	1.515	0.523	8.407	0.004	4.550	1.634	12.669
AP	0.018	0.005	10.566	0.001	1.018	1.007	1.029
VP	0.007	0.005	1.854	0.173	1.007	0.997	1.018
NYHA	0.097	0.263	0.135	0.714	1.101	0.658	1.845
LVES	0.015	0.035	0.190	0.663	1.015	0.948	1.087
LA	−0.008	0.027	0.099	0.753	0.992	0.941	1.045
LVWI	0.004	0.006	0.350	0.554	1.004	0.992	1.016
β-receptor blockers	0.924	0.348	7.074	0.008	2.520	1.275	4.981
AAD	0.801	0.465	2.974	0.085	2.228	0.896	5.540

*AP, atrial pacing; VP, ventricular pacing; ACE-I, angiotensin converting enzyme inhibitor; ARB, angiotensin II receptor blocker; CCB, calcium channel blocker; AAD, antiarrhythmic drugs*.

**Table 5:** ROC curve analysis of AHRE.

**Test variable**	**Area**	**Standard**	***P***	**95% CI**	**The best diagnostic**
		**error**			**cut-off value**
**The area under the curve**					
Age	0.641	0.034	0.000	0.575–0.708	65
AP	0.692	0.032	0.000	0.628–0.755	53.5

The Kaplan–Meier curve analysis revealed that after pacemaker implantation, patients with symptomatic SSS not only had a higher AHRE incidence but also an earlier AHRE occurrence (8.6 months) compared with patients with AVB (10.7 months) (*P* < 0.001, Table 6). The univariate Cox regression analysis revealed that patient age, diagnosis, AP%, and oral administration of β-receptor blocker therapy were risk factors for the AHRE-free duration after pacemaker implantation (Table 7). The multivariate Cox regression analysis revealed that patient age (OR = 1.041; 95% CI, 1.018–1.064; and *P* < 0.001), symptomatic SSS diagnosis (OR = 2.225; 95% CI, 1.227–4.036; and *P* = 0.008), AP% (OR = 1.010; 95% CI, 1.002–1.017; and *P* = 0.016), and oral administration of β-receptor blockers (OR = 1.569; 95% CI, 1.007–2.445; and *P* = 0.047) were risk factors for an early AHRE onset (Table 8).

**Table 6:** Kaplan–Meier analysis of AHREs.

**Diagnosis**	**Mean**				**Median**			
	**Estimate**	**Standard error**	**95% CI**		**Estimate**	**Standard error**	**95% CI**	
			**Lower limit**	**Upper limit**			**Lower limit**	**Upper limit**
**Mean and median survival analysis time**								
Atrioventricular block (cases)	10.672	0.343	10.000	11.345				
Sick sinus syndrome (cases)	8.590	0.330	7.942	9.237	11.000	0.430	10.158	11.842
Overall	9.258	0.256	8.756	9.760	11.000	0.313	10.386	11.614

*Log rank test, X^2^ = 16.056, P < 0.0001*.

**Table 7:** Univariate Cox's regression analysis of AHRE.

**Items**	**B**	**SE**	**Wald**	**Significance**	**Exp (B)**	**95% CI**	
						**Lower limit**	**Upper limit**
Gender	−0.015	0.210	0.005	0.944	0.985	0.652	1.488
Age	0.036	0.011	10.806	0.001	1.037	1.015	1.059
BMI	0.017	0.031	0.293	0.588	1.017	0.956	1.082
Diagnosis	1.017	0.283	12.962	0.000	2.766	1.590	4.812
Hypertension	−0.137	0.213	0.417	0.519	0.872	0.574	1.323
Diabetes	0.215	0.300	0.512	0.474	1.240	0.688	2.234
Coronary heart disease	−0.225	0.373	0.363	0.547	0.798	0.384	1.660
History of TIA/stroke	−0.124	0.512	0.059	0.808	0.883	0.324	2.408
Peripheral vascular diseases	−0.100	0.593	0.029	0.866	0.905	0.283	2.891
CHA_2_DS_2_-VASc	−0.012	0.228	0.003	0.959	0.988	0.633	1.544
NYHA	0.235	0.148	2.517	0.113	1.265	0.946	1.691
LVED	0.015	0.021	0.529	0.467	1.015	0.975	1.057
LVES	0.014	0.020	0.529	0.467	1.014	0.976	1.054
LA	0.015	0.015	0.991	0.319	1.015	0.986	1.045
LVPW	0.107	0.069	2.380	0.123	1.113	0.972	1.274
IVS	0.042	0.050	0.703	0.402	1.042	0.946	1.149
LVEF	−0.007	0.015	0.195	0.659	0.993	0.965	1.023
LVWI	0.006	0.003	3.472	0.062	1.006	1.000	1.013
E/E′	0.013	0.015	0.857	0.355	1.014	0.985	1.043
E/A	0.167	0.226	0.545	0.460	1.182	0.758	1.841
Pacemaker manufacturer	−0.005	0.210	0.001	0.980	0.995	0.659	1.502
AP	0.013	0.003	13.981	0.000	1.013	1.006	1.020
VP	−0.003	0.003	1.180	0.277	0.997	0.992	1.002
ACEI/ARB	0.083	0.231	0.128	0.720	1.086	0.691	1.707
β-receptor blockers	0.657	0.218	9.056	0.003	1.929	1.258	2.960
Statins	−0.126	0.211	0.359	0.549	0.881	0.583	1.332
CCB	−0.070	0.258	0.072	0.788	0.933	0.562	1.548
AAD	0.482	0.265	3.304	0.069	1.619	0.963	2.721
Endpoint event	1.265	0.424	8.889	0.003	3.544	1.543	8.142

*BMI, body mass index; NYHA, New York Heart Association; LVED, left ventricular end-diastolic diameter; LVES, left ventricular end-systolic diameter; LA, left atrial diameter; LVPW, left ventricular posterior wall thickness; IVS, interventricular septal thickness; LVEF, left ventricular ejection fraction; LVWI, left ventricular mass index; AP, atrial pacing; VP, ventricular pacing; ACE-I, angiotensin converting enzyme inhibitor; ARB, angiotensin II receptor blocker; CCB, calcium channel blocker; AAD, antiarrhythmic drugs*.

**Table 8:** Multivariate regression analysis of AHRE.

**Items**	**B**	**SE**	**Wald**	**Significance**	**Exp (B)**	**95% CI**	
						**Lower limit**	**Upper limit**
Age	0.040	0.011	12.214	0.000	1.041	1.018	1.064
Diagnosis	0.800	0.304	6.932	0.008	2.225	1.227	4.036
LVWI	0.003	0.004	0.719	0.396	1.003	0.996	1.010
AP	0.009	0.004	5.780	0.016	1.010	1.002	1.017
β-receptor blockers	0.450	0.226	3.957	0.047	1.569	1.007	2.445
AAD	0.174	0.276	0.396	0.529	1.190	0.693	2.043

*AP, atrial pacing; VP, ventricular pacing; ACE-I, angiotensin converting enzyme inhibitor; ARB, angiotensin II receptor blocker; CCB, calcium channel blocker; AAD, antiarrhythmic drugs*.

## Discussion

The results of the present study revealed a post-PPM-implantation AHRE incidence of 31.5%. Furthermore, patient age, symptomatic SSS diagnosis, PPM implantation, and AP% after PPM implantation were AHRE risk factors. Patients aged >65 years diagnosed with symptomatic SSS with a post-pacemaker-implantation AP% of ≥53.5% required follow-up. Once AHRE occurs, 12-lead synchronous electrocardiography or 12-lead 24-h Holter should be carried out for early AF detection and treatment.

AF is the most common persistent type of arrhythmia in adults; it is related to the increase in incidence and mortality rates of many diseases (2). Certain patients with AF are asymptomatic, which leads to clinical missed diagnoses. CIEDs with atrial leads can detect atrial arrhythmia events, including atrial tachycardia (AT), AFL, and AF. These events are usually asymptomatic and can only be detected by CIEDs through long-term continuous heart rate monitoring (8). Patients implanted with ICD, CRT-P, and CRT-D usually have underlying diseases, which will increase the incidence of atrial arrhythmia (1). Kleemann et al. (9) revealed that more than half of primary prophylactic ICD patients with sinus rhythm at baseline develop new AF or ventricular tachycardia or ventricular fibrillation after 6 years. Gonzales et al. (5) reported that previous heart failure was a AHRE predictor, and Wilton et al. (10) revealed that in trials on resynchronization in ambulatory heart failure, AF/AT after implantation of CRT/D was detected in nearly half of the patients after randomized grouping. Therefore, the present study only included patients with PPM implantation and excluded patients with ICD or CRT implantation.

At present, the AF prevalence in adults is 2–4%. With the increase in life expectancy and the widespread development of AF screening in the population, it is expected that the prevalence of AF will increase by 2.3 folds in the future (2). Patients with a previous history of AF were excluded from the present study. AHRE was detected in 91/289 patients who completed the 12-month follow-up. The AHRE incidence was 31.5%, which is similar to the incidence reported in relevant literature (8) but much higher than the AF prevalence in the general population. There are several possible reasons for this: (1) AF is diagnosed using electrocardiography; it is easy to miss the diagnosis when patients with paroxysmal AF are asymptomatic with a low frequency and short duration. AHRE is the result of continuous detection via CIEDs. Therefore, after CIED implantation, the AHRE detection rate is significantly higher than the AF detection rate (1); (2) most AHREs are asymptomatic or short-term AF, AFL, or AT. However, when other tachyarrhythmias, such as sinus tachycardia, paroxysmal supraventricular tachycardia, or ventricular tachycardia with 1:1 atrioventricular retrograde conduction, reach the AHRE diagnostic criteria (atrial rate ≥ 175 bpm, duration ≥ 5 min), they will be diagnosed as AHREs by the CIEDs. Most studies use ≥5 min to define AHRE; however, they have a false-positive rate of 17.3% (11); and (3) a false-positive AHRE diagnosis can also be caused by atrial pacing lead over sensing, far-field R wave sensing, pacing lead-mediated arrhythmia, or other external signal interferences (12). Although Bertaglia et al. (8) believe that AHREs lasting >5–6 min are the “diagnostic sweet spot,” it enables most AHRE detection algorithms to distinguish real atrial arrhythmia from external signal interference. The results of the present study confirmed that AHRE-positive patients mostly had AF/AFL; however, the diagnosis may also be a false positive or SVT and other tachyarrhythmias, and AHRE-positive patients cannot be simply considered to have AF/AFL. Further surface electrocardiography or Holter examinations should be performed to eliminate false positives.

Age is not only the most important risk factor of AF (2), but also AHRE (6). In the present study, the patient age was significantly higher in group A than in group N. The multivariate logistic regression analysis revealed that patient age (OR = 1.060; 95% CI, 1.027–1.093; and *P* < 0.001) was an AHRE risk factor; the older the patient, the higher the risk of AHRE. The ROC curve analysis revealed that the best diagnostic cut-off value was 65 years (AUC = 0.641; sensitivity = 74.7%; specificity = 47.5%; and *P* < 0.001).

Both sinoatrial nodal dysfunction (SND) and AF are associated with atrial remodeling. The common pathological change is atrial fibrosis, which results in an extensive low voltage area and slow conduction velocity in the atrium (13). A recent study revealed that *Paired-like homeodomain 2*, the first common AF gene locus, is not only involved in the development of the pulmonary vein (14) but also related to the development of sinoatrial nodes and the asymmetry of the right and left atrium (15). Among patients with SND, 40–70% have atrial arrhythmias, such as AF (15). The results of the ASSERT (The Asymptomatic Atrial Fibrillation and Stroke Evaluation in Pacemaker Patients and the Atrial Fibrillation Reduction Atrial Pacing Trial) (3) revealed that SND and the resting heart rate decrease were AHRE predictors. In their study, Kim et al. (16) enrolled 880 patients with pacemaker implants and no previous history of AF and followed up with them for 7 years. The results revealed a new AF onset in 122 (13.8%) patients. Moreover, the diagnosis of symptomatic SSS and pacemaker implantation were independent risk factors for AF (HR = 2.33; 95% CI, 1.62–3.55; and *P* < 0.001). Kim et al. (11) reported a correlation between symptomatic SSS and AHREs with a duration of >6 min (OR = 3.85; 95% CI, 2.42–6.14; and *P* < 001). The results of the present study revealed that the proportion of patients with pacemaker implantation due to symptomatic SSS was higher in group A (83.5%) than in group N (56.1%) (*P* < 0.001). The multivariate logistic regression analysis revealed that pacemaker implantation based on a symptomatic SSS diagnosis was an AHRE risk factor (OR = 4.550; 95% CI, 1.634–12.669; and *P* = 0.004); this is consistent with the results of Kim et al. (16) and supports the view that symptomatic SSS is closely related with AHRE.

Atrial pacing increases the occurrence of AHREs; Adelstein and Saba (17) revealed that, after CRT implantation, the risk of AF increased 2 folds in patients with atrial pacing than in patients with atrial perception. Fontenla et al. (18) reported that atrial rate-responsive pacing increased the incidence of persistent AF/AT in patients with ICD implants (OR = 3.58; 95% CI, 1.82–7.03; and *P* < 0.001). In the present study, the AP% was significantly higher in group A than in group N. The multivariate logistic regression analysis revealed that AP% (OR = 1.010; 95% CI, 1.002–1.017; and *P* < 0.016) was an AHRE risk factor; the higher the AP%, the higher the risk of AHRE. The ROC curve analysis revealed that the best AP% diagnostic cut-off value was 53.5% (AUC = 0.692; sensitivity = 69.2%; specificity = 63.1%; and *P* < 0.001).

A previous study revealed that left atrial enlargement was not only closely related to the occurrence and development of AF but also a predictor of AF recurrence after radiofrequency ablation (19). Atrial enlargement is accompanied by different degrees of atrial fibrosis, and that atrial fibrosis may be an important characteristic of persistent AF (19). Kim et al. (11) revealed that left atrium enlargement (>41 mm) was associated with AHRE occurrence (OR = 1.96; 95% CI, 1.00–3.85; and *P* = 0.050). The present study also revealed that the left atrium was significantly larger [38.0 mm [33.8–41.9 mm]] in group A than in group N [35.6 mm [32.8–38.9 mm]] (*P* = 0.009). These results suggest that left atrial enlargement is closely related with AHRE occurrence. A number of clinical diseases (e.g., hypertension, coronary heart disease, heart failure, cardiomyopathy, obesity, and diabetes) can induce atrial fibrosis; this suggests that in AF treatment, attention should also be paid to comprehensively treating patient complications. This is consistent with the guidelines' treatment path (2).

The results of the present study suggest that the proportion of patients who underwent an oral administration of β-receptor blockers or had AADs (or both) was significantly higher in group A than in group N (*P* < 0.001). This may be due to the occurrence of AHRE-induced clinical symptoms, such as palpitations and chest tightness, in group A, and clinicians prescribing more of the above-stated drugs, such as amiodarone, propafenone and sotalol, for treatment, so, AHRE is the cause of using AADs or β-receptor blockers.

The present study has the following limitations: (1) it is a single-center retrospective case study, the sample size is small, and there may be a certain degree of bias; (2) cases of CRT or ICD implantation were excluded and, thus, the AHRE detection rate may be underestimated; (3) it has a 12-month follow-up period and does not involve the incidence of stroke, systemic embolism, heart failure, acute myocardial infarction, and other cardiovascular and cerebrovascular events within 12 months of pacemaker implantation. In the follow-up study, we will explore the direct correlation between AHREs and clinical cardiovascular and cerebrovascular events, along with the risk factors.

## Data Availability Statement

The raw data supporting the conclusions of this article will be made available by the authors, without undue reservation.

## Ethics Statement

This study was conducted with approval from the Ethics Committee of Fujian Medical University Union Hospita (No:2021KY047). The patients/participants provided their written informed consent to participate in this study.

## Author Contributions

HZ, QC, and FF conceived the idea and conceptualized the study. FZ, LC, and WW collected the data. HZ analyzed the data. JC, GC, and WW drafted and reviewed the manuscript. All authors read and approved the final draft.

## Funding

This research was supported by the Guiding project of Fujian science and Technology Department (No: 2017Y0039).

## Conflict of Interest

The authors declare that the research was conducted in the absence of any commercial or financial relationships that could be construed as a potential conflict of interest.

## Publisher's Note

All claims expressed in this article are solely those of the authors and do not necessarily represent those of their affiliated organizations, or those of the publisher, the editors and the reviewers. Any product that may be evaluated in this article, or claim that may be made by its manufacturer, is not guaranteed or endorsed by the publisher.
